# Pattern Recognition and Functional Neuroimaging Help to Discriminate Healthy Adolescents at Risk for Mood Disorders from Low Risk Adolescents

**DOI:** 10.1371/journal.pone.0029482

**Published:** 2012-02-15

**Authors:** Janaina Mourão-Miranda, Leticia Oliveira, Cecile D. Ladouceur, Andre Marquand, Michael Brammer, Boris Birmaher, David Axelson, Mary L. Phillips

**Affiliations:** 1 Department of Computer Science, University College London, United Kingdom; 2 Department of Neuroimaging, King's College London, United Kingdom; 3 Instituto Biomédico, Universidade Federal Fluminense, Niteroi, Brazil; 4 Department of Psychiatry, Pittsburgh University, Pittsburgh, Pennsylvania, United States of America; 5 Department of Psychological Medicine, Cardiff University, Cardiff, United Kingdom; University of Leuven, Belgium

## Abstract

**Introduction:**

There are no known biological measures that accurately predict future development of psychiatric disorders in individual at-risk adolescents. We investigated whether machine learning and fMRI could help to: 1. differentiate healthy adolescents genetically at-risk for bipolar disorder and other Axis I psychiatric disorders from healthy adolescents at low risk of developing these disorders; 2. identify those healthy genetically at-risk adolescents who were most likely to develop future Axis I disorders.

**Methods:**

16 healthy offspring genetically at risk for bipolar disorder and other Axis I disorders by virtue of having a parent with bipolar disorder and 16 healthy, age- and gender-matched low-risk offspring of healthy parents with no history of psychiatric disorders (12–17 year-olds) performed two emotional face gender-labeling tasks (happy/neutral; fearful/neutral) during fMRI. We used Gaussian Process Classifiers (GPC), a machine learning approach that assigns a predictive probability of group membership to an individual person, to differentiate groups and to identify those at-risk adolescents most likely to develop future Axis I disorders.

**Results:**

Using GPC, activity to neutral faces presented during the happy experiment accurately and significantly differentiated groups, achieving 75% accuracy (sensitivity = 75%, specificity = 75%). Furthermore, predictive probabilities were significantly higher for those at-risk adolescents who subsequently developed an Axis I disorder than for those at-risk adolescents remaining healthy at follow-up.

**Conclusions:**

We show that a combination of two promising techniques, machine learning and neuroimaging, not only discriminates healthy low-risk from healthy adolescents genetically at-risk for Axis I disorders, but may ultimately help to predict which at-risk adolescents subsequently develop these disorders.

## Introduction

Early identification of individuals at high risk of future psychiatric illness is a critical but challenging endeavour. Because most psychiatric disorders typically have an onset in adolescence or early adulthood [1], meeting this challenge could delay, or even prevent, future onset of these debilitating illnesses in high-risk adolescents. To date, no biological measures can either accurately identify individual risk for future psychiatric illness, or predict future illness onset *at an individual level*: even genetic risk does not accurately predict *individual risk* for future psychiatric illness. For example, having a family history of bipolar disorder confers a 10% risk of future bipolar disorder, as well as 10–25% risk of developing other Axis I disorders in the future, including ADHD, major depression, or anxiety disorders [2]. Having such family history does not, however, accurately determine the likelihood of future development of these disorders at the individual level.

Two techniques: machine learning and neuroimaging, show promise as tools to identify biological measures that may help with clinical challenges such as early identification of individuals at future risk of psychiatric disorders. Neuroimaging techniques such as functional magnetic resonance imaging (fMRI) have helped improve understanding of abnormalities in neural circuitry supporting emotion processing and emotion regulation associated with a variety of different psychiatric disorders, including mood disorders such as bipolar disorder and major depressive disorder, in adults [3], [4], [5] and adolescents [6], [7]. Furthermore, neuroimaging and univariate statistical techniques have been used to indentify circuitry abnormalities in adolescents at risk of future mood disorders as group differences relative to a healthy control group. These techniques cannot, however, be used to identify robust abnormalities in neural circuitry *in individual participants*; nor can such techniques be used to classify individuals into diagnostic groups based upon neuroimaging findings. Machine learning comprises computer-based techniques that allow automatic discovery of regularities in data (i.e. patterns). Discovery of such regularities can then be used to classify data into different categories. Machine learning has previously been applied to classify groups of individuals based on structural MRI data [8], [9], and can also be applied to functional magnetic resonance imaging (fMRI) data to classify *individuals*, case by case, into groups based on their fMRI data [10], [11]. For example, the combination of machine learning with fMRI has recently been used to accurately differentiate depressed patients from healthy individuals on a case-by-case basis [11]. These techniques are therefore promising tools to be used for clinical purposes, such as identification of neurobiological measures that can aid early identification of those individuals who are at future risk of developing psychiatric disorders [12], , but their potential in this domain remains unrealized.

In the present study, we sought to determine whether machine learning and fMRI could help to discriminate, at an individual level, healthy adolescents genetically at-risk for Axis I disorders including bipolar disorder, depression, and anxiety disorders, from healthy adolescents at low risk of developing these disorders. In more exploratory analyses, we also investigated whether the predictive probabilities of those healthy, genetically at-risk adolescents who subsequently developed an Axis I disorder, were statistically different from the predictive probabilities of those who did not develop such a disorder in follow-up clinical assessments. To achieve this, we used a well-validated emotional face gender labeling fMRI paradigm [14]. This type of experimental design has been used to examine neural activity in mood disorders given that abnormal patterns of neural activity during facial emotion processing has been shown in a range of psychiatric conditions, especially in depression and bipolar disorder [5], [6].

## Methods

### Participants

The study was approved by the University of Pittsburgh Institutional Review Board. Parents signed consent forms, and adolescents signed assent forms. A total of 32 healthy offspring participated in the study (Table 1). Of these, 16 were offspring having at least one biological parent diagnosed with bipolar disorder, who were therefore at genetic risk of future psychiatric disorders (healthy bipolar offspring). 16 offspring were age- and sex- matched healthy offspring of healthy parents (healthy controls). Parents of healthy controls did not have any current Axis I psychiatric disorder or history of mood disorder or psychotic disorder, while first-degree relatives of healthy controls did not have any current, or history of, bipolar disorder. Participants were recruited from the Bipolar Offspring Study (BIOS), an ongoing longitudinal study on the psychiatric symptomatology in offspring of parents with bipolar disorder (MH#060952, PI: B.B.).

**Table 1:** Demographic and Clinical Characteristics of Healthy Offspring Having a Parent with Bipolar Disorder and Age- and Sex- Matched Control Offspring of Healthy Parents.

	Group		Statistic	df	*p* Value
	HBO (n = 16)	HC (n = 16)			
Age at Scan (years), mean (SD)	14.8 (1.8)	15.3 (1.2)	t = 1.2	30	.25
Sex (M/F)	7/9	7/9	—	—	—
Socio-economic Status, mean (SD)	45 (13)	44 (11)	t = −.57	21	.83
Full Scale IQ, mean (SD)	118 (12)	121 (11)	t = −.57	15	.57
MFQ – parent version, mean (SD)	3.6 (4.1)	2.7 (5.5)	t = .54	30	.59
MFQ – child version, mean (SD)	7.4 (5.7)	4.6 (3.9)	t = 1.6	29	.13
SCARED – parent version, mean (SD)	6.4 (5.0)	4.6 (5.3)	t = 1.6	30	.33
SCARED – child version, mean (SD)	14.1 (9.6)	9.6 (6.0)	t = .99	30	.12
CALS, mean (SD)	3.6 (3.9)	2.8 (4.6)	t = .54	30	.60

Abbreviations: HBO = healthy offspring having a parent diagnosed with bipolar disorder; HC = healthy control offspring of healthy parents; MFQ, Mood and Feelings Questionnaire (range, 0–68); SCARED, Screen for Childhood Anxiety and Related Disorders (range, 0–82); CALS, Child Affect Lability Scale (range, 0–80).

Procedures for the above longitudinal study included diagnostic interviews with the offspring and their parents using semi-structured diagnostic instruments: The Structural Clinical Interview for DSM-IV (SCID-I) was used to ascertain lifetime psychopathology for all parents and the Schedule for Affective Disorders and Schizophrenia for School Aged Children – Present and Lifetime Version (K-SADS-PL) [15] was used to interview parents about their children and children about themselves for the presence of current and lifetime psychiatric disorders (see [2] for details). The family history–research diagnostic criteria method [16] was used to ascertain the psychiatric history of biological co-parents not seen for direct interview.

Participants in the above longitudinal study who did not endorse any current DSM-IV Axis I diagnosis or history of depression or bipolar disorder on the K-SADS-PL were invited to participate in the current neuroimaging study. Participants and their parents completed self-report measures on the day of the neuroimaging scan to ensure that all participants were free of any current DSM-IV Axis I psychiatric diagnoses immediately before the neuroimaging evaluation. Parents completed self-reports about their children assessing: presence of DSM-IV Axis I disorder, anxiety, depression, and mood lability. Children completed self-report measures assessing anxiety and depression (see Text S1). Exclusion criteria included: IQ<70, history of head trauma, neurological disorder, substance abuse/dependence, developmental delay, hand-eye coordination problems, and mood disorders secondary to substance abuse, medical conditions, pregnancy, presence of metal in the body.

### fMRI paradigm

An emotional face gender labeling event-related fMRI paradigm was used [14]. It comprised two, well-validated 6-minute fast event-related neuroimaging tasks examining neural activity to happy versus neutral (happy face task) and fearful versus neutral (fearful face task) emotional facial expression. Faces were morphed to depict expressions ranging from neutral, to mild (50%), and to intense (prototypical; 100%). Subjects were asked to indicate whether the actor in the picture was a woman or a man (see Text S1).

### Image acquisition

Neuroimaging data were collected using a 3T Siemens Allegra MRI. The acquisition parameters are described in Text S1.

### Data preprocessing and analysis

Data preprocessing was performed using standard procedures in SPM5 (see Text S1 for details). For each subject a general linear model (GLM) was constructed in SPM5 with three emotion intensities (e.g., neutral, mild happy, intense happy) entered in the design matrix as separate regressors. The happy face and fearful face tasks were modeled separately. The fixation cross served as a baseline. Movement parameters from the realignment stage were entered as covariates of no interest to control for subject movement. The images corresponding to the GLM coefficients for each experimental condition (neutral, mild happy/fearful, intense happy/fearful) defined the spatial patterns of brain activation used as input to the Gaussian Process Classifier (GPC).

### Pattern Classification Analysis

We used Gaussian Process Classifiers (GPCs) [17], a machine learning approach that assigns a predictive probability of group membership to an individual person based on the confidence of a classifier computed from pre-processed fMRI scans. For a detailed description about the GPC implementation to fMRI based classification please see [18]. We used a Gaussian process classifier (GPC), as the test predictions take the form of predictive probabilities; this contrasts with other classification methods that provide categorical classification (e.g. +1 for class 1 and −1 for class 2). The predictive probability measures the classifier confidence about the class membership of the test example. Probabilistic predictions are especially important for clinical applications for two reasons. First, probabilistic prediction models aim to provide coherent estimates of predictive uncertainty for individual subjects (e.g. the probability that a particular subject has a psychiatric disorder). In clinical populations, it is reasonable to expect that illness severity varies within patient groups and the disease itself may also be heterogeneous. Probabilistic predictions provide an elegant mechanism to capture this variability, providing confident predictions for the most prototypical cases and less confident predictions for more ambiguous cases. Second, probabilistic predictions can be easily adjusted to compensate for the prior frequency of diagnostic classes in the general population [10]. Thus, probabilistic prediction models provide mechanisms to ensure that inference remains coherent in classification scenarios where the frequency of each class in the test set may be entirely different from the frequencies observed in the training set.

**Figure 1:**
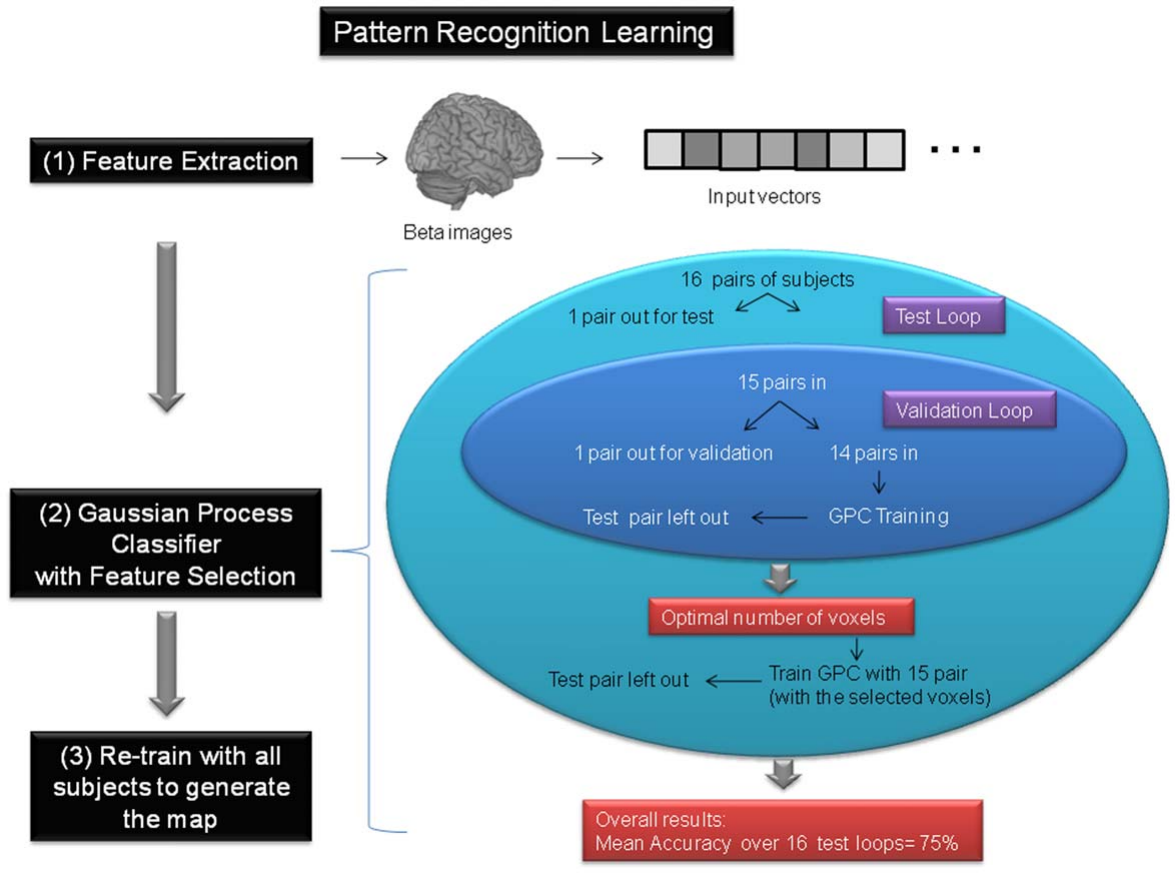
Summary of pattern recognition analyses. (1) Feature Extraction: the information from the beta images were transformed into an input vector. (2) Nested leave one out (LOO) Approach. We employed a nested (3-way) cross-validation, where we first excluded one matched pair of subjects to comprise the test set (test loop in light blue). We then performed a second split (validation loop in dark blue), where we removed 5000 voxels each iteration and repeatedly repartitioned the remaining 15 subject pairs into a validation set (1 pair) and training set (14 pairs) to compute the mean accuracy on the validation set. This procedure (removing voxels and computing mean accuracy) was repeated until all voxels were removed. We then selected the number of voxels that produced maximal accuracy on the validation set before applying it to the test set. The final accuracy was the mean accuracy over all test subjects (outer test loop in light blue). (3) We then generated a map training the GPC with all subjects and removing voxels until we obtained the mean number of voxels.

We used GPC as implemented in the PROBID software package (http://www.brainmap.co.uk/PROBID). We embedded the classifier in a recursive feature elimination (RFE) framework [18], a well-validated technique in pattern recognition analyses, that enabled us to: (1) find the subset of brain voxels that provided the optimal discrimination accuracy and (2) accurately localize the most discriminative brain voxels. To achieve this, we employed nested (3-way) cross-validation where we first excluded a matched pair of subjects (one from each group) to comprise the test set, then performed a second split where we repeatedly repartitioned the remaining 15 subject pairs into a validation set (1 pair) and training set (14 pairs). In each case we selected the number of features that produced maximal accuracy on the validation set before applying it to the test set (Figure 1). We thresholded the probabilistic predictions at 0.5 to convert the probabilistic predictions to class labels and computed the proportion of subjects having the correct label across all test splits to derive an overall estimate of generalization ability (classification accuracy). The statistical significance of the classifier was determined by permutation testing.

### Follow-up clinical assessment

As part of their enrollment in the longitudinal study, each participant was interviewed *face-to-face* every other year using the same procedures and instruments utilized during the intake interview described above (see [2]). Presence or absence of Axis I disorder at the follow-up clinical interview, which was conducted every other year, was used in analyses. Given that the longitudinal follow-up study is still ongoing, the most recent information available was included in the current analyses. The mean interval of time between the fMRI scan and the follow-up interview was 24.4 months (range 12 to 45 months).

### Receiver Operating Characteristic curve

Using the predictive probabilities from the classifier for at-risk adolescents versus healthy controls as a score for at-risk adolescents, we were then able to use receiver operating characteristic (ROC) curve analysis to evaluate if this score could be used to predict which at-risk adolescents developed a psychiatric disorder during follow-up clinical assessment. The ROC curve was constructed using the predictive probabilities for all at-risk adolescents with follow-up, and the labels were developers vs. non-developers. The ROC curve compared the classifier's true positive rate (TP) and false positive rate (FP) as the decision threshold (i.e. the score threshold) was varied. A classifier guessing at chance level would therefore result in an 45 degree diagonal line that connects the point (0, 0) with the point (1,1), while classifiers discriminating above chance would result in an ROC curve that is ‘northwest’ of this line. The area under the curve (AUC) is therefore a summary measure describing the performance of the classifier across all decision thresholds, where a classifier achieving perfect classification would achieve an AUC of 1, while a classifier guessing at chance-level would achieve an AUC of 0.5.

### Permutation test

This test was used to derive a p-value to determine whether classification accuracy exceeded chance levels (50%). To achieve this, we permuted the class labels 1000 times (i.e., each time randomly assigning class 1 and class 2 labels to each pattern of brain activation) and repeated the entire RFE procedure. We then counted the number of times the permuted test accuracy was higher than the one obtained for the true labels. Dividing this number by 1000 we derived a p-value for the classification accuracies. We also performed the permutation test for the area under ROC curve.

### Additional Measures

#### Behavioral performance on the fMRI paradigm

Mean percent accuracy scores and correct-trial reaction times were analyzed using mixed analyses of variance models, with group as between-subject and face condition as within-subject variables.

#### Post-scanning emotion labeling task

Following the scanning session, participants performed a computerized emotion labeling task. The task comprised viewing 45 grayscale pictures of male and female actors [19] depicting several different emotional facial expressions (happy, sad, anger, fear, disgust, neutral). Participants were asked to select the appropriate emotion label by using a mouse to click on the square next to the emotional word. For the purpose of this study, analyses focused on overall accuracy scores and reaction times, and specifically for happy, fearful, and neutral faces.

## Results

### Pattern Classification

#### Happy face task

**Figure 2:**
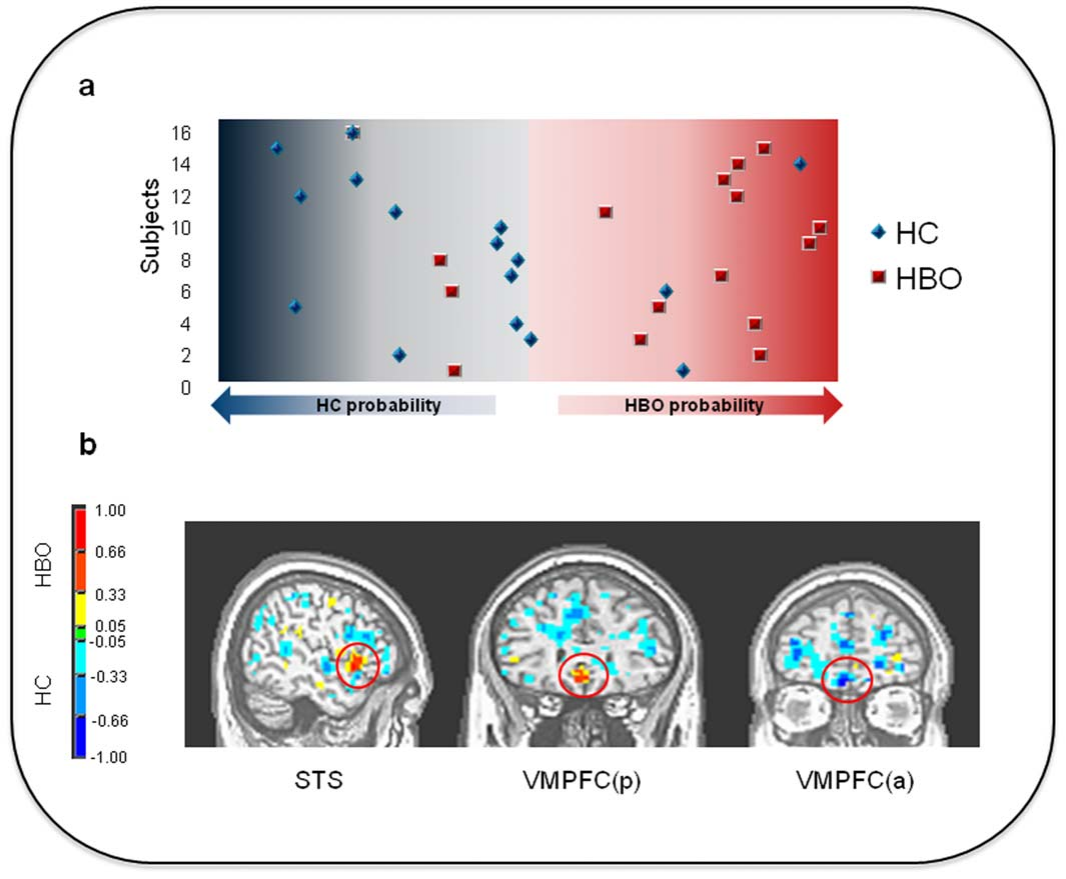
Summary of results from pattern recognition analyses. A. Decision boundary and individual predictive probabilities. B. GPC weights overlaid on an anatomical template. The color code shows the relative weight of each voxel for the decision boundary (red scales: higher weights for healthy bipolar offspring and blue scales: higher weights for healthy controls). The discriminating pattern included clusters with higher weights for healthy bipolar offspring in the superior temporal sulcus (STS; x, y, z: -50, 11, -5) and in a posterior region of the ventromedial prefrontal cortex (VMPFC(p); x, y, z,: 0, 29, -14) and a cluster with higher weights for healthy controls in the anterior region of the ventromedial prefrontal cortex (VMPFC (a); x, y, z: -2, 51, -19) (x, y, z, are in Talairach coordinates).

GPC based on the whole brain activity to neutral faces accurately and significantly differentiated at-risk adolescents from healthy controls with 75% accuracy (sensitivity = 75%, specificity = 75%, permutation test p = 0.008). Only 4/16 at-risk adolescents were misclassified as healthy controls (Figure 2A). For the mild faces, the accuracy in differentiating groups was 68.5% (specificity = 56%, sensitivity = 81%, permutation test p = 0.07). For the intense faces, the accuracy was only 37.5% (sensitivity = 44%, specificity = 31%, permutation test p = 0.96). The spatial pattern that best discriminated the groups included ventromedial prefrontal cortex and superior temporal sulcus (Figure 2B, see also Table S3 for the list of regions). We emphasize that the discrimination maps should not be interpreted as statistical tests; they simply provide a spatial representation of the decision boundary, i.e. the weight of each voxel in discriminating the groups. In the present study, we used Recursive Feature Elimination (RFE) to select a subset of most important regions for discriminating the groups. Gaussian Process Classifiers are multivariate techniques, however, and therefore take into account spatial correlations in the data. Since the discrimination is based on the whole pattern, rather than on individual regions, all voxels within the pattern contribute the classification and no local inferences based on these approaches should be made.

#### Fearful face task

Whole-brain GPC classification accuracy did not significantly exceed chance for neutral, mild or intense faces. Specifically, for neutral faces the accuracy was 47% (sensitivity = 63%, specificity = 31%), to mild faces the accuracy was 40.5%(sensitivity = 50%, specificity = 31%), and to intense faces, was 66% (sensitivity = 88%, specificity = 44%).

#### Outcome

**Figure 3:**
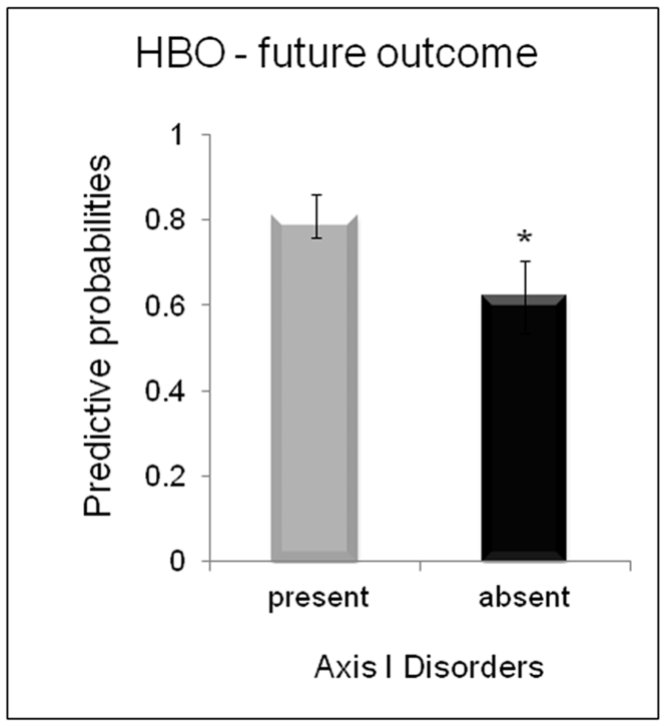
Mean predictive probabilities (with standard error to the mean) for the comparison between healthy bipolar offspring with and without future onset of Axis I disorder, which in this sample was major depressive disorder and anxiety disorders.

The predictive power of the GPCs for future Axis I disorder in at-risk adolescents pertaining to neutral faces presented during the happy face experiment was evaluated using post-scanning clinical assessments in 13 at-risk adolescents. Of these, 6 subsequently met DSM-IV criteria for either major depression (n = 3) or anxiety disorders (n = 3). GPC predictive probabilities were significantly higher for these 6 at-risk adolescents than for at-risk adolescents who remained healthy at follow-up (t(11) = 1,82, p = 0.04) (Figure 3). Furthermore, 3/4 at-risk adolescents misclassified as healthy controls at scanning remained healthy at follow-up (for one of these 4 at-risk adolescents, we did not have clinical follow-up information).

#### ROC Analyses

**Figure 4:**
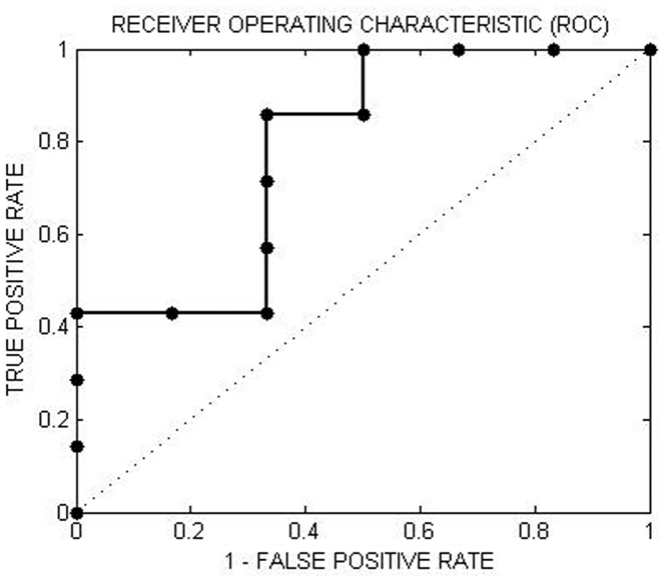
We used the predictive probabilities from the classifier at-risk adolescents vs. controls as a score for the at-risk adolescents. An ROC curve was used to evaluate if this score could be used to predict which of at-risk adolescents developed a future mood disorder. Each point on the ROC curve represents a sensitivity/specificity pair corresponding to a particular decision threshold. A test with perfect discrimination has a ROC curve that passes through the upper left corner (100% sensitivity, 100% specificity). The area under the ROC curve (AUC) was 0.78 (p<0.05).

The area under the ROC curve (AUC) was 0.78 (p<0.05, permutation test) (Figure 4), indicating that the score based on the classifier for at-risk adolescents versus healthy controls could be used to predict those at-risk adolescents who went on to develop, versus those who did not develop, a psychiatric disorder during clinical follow-up (i.e. area under the ROC curve exceed chance level which is 0.5). Using a combination of machine learning and neuroimaging, we were therefore able to find a measure (i.e. GPC predictive probabilities) that could be used to identify which at-risk adolescents subsequently developed an Axis I disorder.

### Task performance on fMRI paradigm

#### Happy Face Task: Accuracy

There was no significant main effect of group F(1,30) = 2.4, *p* = .14), or group by face condition interaction (F(2,29) = 0.01, *p* = .99). There was, however, a significant main effect of face condition (F(2,29) = 15.8, *p*<.001), indicating that percent accuracy was significantly lower for neutral faces relative to happy faces (mild, intense), *p*<.05. *Reaction times*: There was no significant main effect of group, main effect of face, or group by face condition interaction (all p>.1).

#### Fearful Face Task: Accuracy

There was no significant main effect of group (F(1, 30) = 0.99, *p* = .33), or group by face condition interaction (F(2,29) = 0.50, *p* = .61). There was, however, a significant main effect of face condition, (F(2,29) = 11.2, *p*<.001), indicating that percent accuracy was significantly lower for neutral faces relative to intense fearful faces (*p*<.05). The effect was at a trend level for mild fearful versus neutral faces (p = .06). *Reaction times*: There was no significant main effect of group, main effect of face, or group by face condition interaction (all p>.1) (see Table S1).

### Task performance on post-scanning emotion labeling task

#### Accuracy

There were no significant group differences on overall emotion labeling scores (t(23) = .92, *p* = .37). When examining accuracy specifically to happy, fearful, and neutral faces, however, there was a main effect of face condition (F(2,22) = 11.10, *p*<.001). Post hoc comparisons indicated that accuracy scores were significantly lower to neutral faces relative to happy and fearful faces (*p*<0.05).

#### Reaction times

There were no significant group differences on emotion labeling scores (t(23) = .45, *p* = .66). When examining reaction times specifically to happy, fearful, and neutral faces, however, there was a main effect of face condition (F(2,22) = 6.23, *p* = .007). Post hoc comparisons indicated that reaction times were slower to fearful relative to happy and neutral faces, (p<.05; see Table S2).

## Discussion

The main goal of this study was to determine whether machine learning and fMRI could help to differentiate, at an individual level, healthy adolescent offspring at genetic risk for bipolar disorder and other Axis I psychiatric disorders from healthy adolescents at low risk of developing bipolar disorder or other Axis I psychiatric disorders. We also investigated whether the predictive probability of those healthy at-risk adolescents who subsequently developed a future psychiatric illness were statistically different from the predictive probability of those at-risk adolescents who did not develop such a disorder in longitudinal clinical follow up. Our findings indicate that machine learning combined with fMRI helped to discriminate healthy low-risk control adolescents from healthy adolescents at genetic risk of future psychiatric disorders. Our findings also indicate that the magnitude of the predictive probabilities for group classification that were derived from these techniques could potentially be used as a score to predict which at-risk adolescents subsequently went on to develop an Axis I psychiatric disorder, namely mood and anxiety disorders.

The advantage of pattern recognition techniques such as the one we employed in the present study is that they provide information at the individual – rather than the group - level (GPC based on whole brain neuroimaging data). Specifically, our findings from GPC indicate that we can make predictions at the individual level considering the discrimination between who are genetically at risk from who are not. Furthermore, ROC analysis with predictive probabilities derived from GPC suggests that pattern recognition techniques such as GPC have the potential in the future to help identify which at-risk adolescents are most likely to develop future Axis I disorders.

Recent studies demonstrated the utility of pattern recognition approaches in helping with classification of different psychiatric disorders, including Alzheimer's diseases and autism [8], [9]. One study [20] evaluated early recognition and disease prediction using multivariate pattern classification, and demonstrated that this approach could be used to predict transition to psychosis. Until now, however, it was unknown whether the method could distinguish completely asymptomatic, genetically at-risk individuals from healthy, low-risk individuals.

In the present study, none of the at-risk adolescents developed bipolar disorder following the fMRI scan. Over half of the at-risk adolescents developed an affective disorder (anxiety or depression), however. It is possible that the prediction findings in the present study may be attributed to similarities across affective disorders with regard to abnormal patterns of activation to emotional facial expressions relative to age-matched healthy controls. However, the fact that healthy at-risk adolescent who went on to develop these disorders have higher predictive probabilities is noteworthy in light of evidence indicating that major depressive disorder and anxiety disorders often emerge prior to the onset of mania and episodes of depression characterizing bipolar disorder [1]. For example, recent evidence from the Bipolar Offspring Study suggests that a larger number of bipolar disorder episodes of offspring in that study started with depressive episodes [2]. Such evidence supports previous findings indicating that depressive symptoms emerge prior to the onset of bipolar disorder symptoms [21], [22], [23], particularly if there is family history of bipolar disorder [24]. With regard to anxiety disorders, some studies suggest that anxiety symptoms often precede and may hasten the onset of bipolar disorder in adults [25]. For example, prospective data from a large community sample suggested that individuals who reported experiencing anxiety as adolescents were at increased risk of developing bipolar disorder as adults [26], which is consistent with previous findings [27], [28]. Ongoing prospective follow-up of the at-risk adolescents in the current study will help to further elucidate the role of these psychiatric disorders in the developmental course of bipolar disorder. Identifying differences in patterns of neural activity to emotionally salient information in these at-risk adolescents can contribute valuable information to this research.

Gaussian process classifiers are discriminative approaches and therefore are able to find a discriminating boundary between two classes (e.g. healthy vs. a patient group), and then use this information to classify new individuals. Discriminative models, however, should not be confused with statistical approaches based on mean group differences, such as the General Linear Model [29]. GLM analyses treat every voxel independently and extract measures of interest from them, such as the average response during a particular experimental condition. More specifically, the GLM approach searches for voxels whose activation time series is well reconstructed by the combination of regressor time series related with each experimental condition and some noise terms. The analysis of fMRI data with discriminative models differs from the traditional GLM analysis by investigating a different question. Instead of finding voxels whose time series respond to a specific experimental condition, such models ask whether it is possible to make a prediction about a variable of interest (e.g. patients vs. controls or task 1 vs. task 2) based on the pattern of activation over a set of voxels [30]. Furthermore, discriminative models provide a map that shows the discriminating *boundary* between the different groups. Specifically, a high value in a particular voxel indicates a strong contribution to the discrimination boundary, but does not necessarily imply greater activity in one group versus another. In summary, pattern recognition approaches such as GPC are multivariate techniques, where discrimination is based on the whole pattern rather than on regional activity, which is typically reported in the traditional GLM-based analyses comparing psychiatric patient and healthy control groups. Based on the GPC used in the current study, the spatial pattern that best discriminated at-risk adolescents vs. healthy controls included ventromedial prefrontal cortex and superior temporal sulcus, which are key regions supporting emotion regulation and face processing, and are regions that have been shown to be functionally abnormal in individuals with bipolar, and other mood disorders [3], [31].

Interestingly, the best discrimination between at risk and low-risk adolescents was found to be neutral faces presented in the happy face experiment. Furthermore, the fact that there were no significant findings for mild or intense happy faces, or for any of the faces in the fear face experiment, suggested that accurate classification was specific to neutral faces presented in the context of happy faces, and not generalizable to the other emotional faces. Neutral faces especially are often perceived as ambiguous and potentially threatening by individuals diagnosed with anxiety or mood disorder [6], [32]. One study, for example, reported abnormally elevated subcortical activity to neutral faces in youth with bipolar type I disorder, particularly in those who perceived these faces as threatening [6]. Another study reported that depressed patients did not differ from healthy controls in their ability to accurately recognize sad and happy facial expressions, but they were less accurate at recognizing neutral expressions [33]. Specifically, depressed patients misclassified a higher number of neutral expressions as sad, suggesting a negative interpretative bias. Other studies used neutral facial expressions as a control condition for other emotional facial expressions, and also found evidence of a negative emotional interpretative bias in depressed patients [34], or found that depressed patients were slower to respond to neutral expressions compared with emotional expressions [33].

Findings from the behavioural data analyses indicate that there were no differences between groups, or group by face condition interactions, neither in accuracy nor for reaction times. This is interesting because it shows that behavioural differences cannot explain the results found in the present study, indicating that the classification results are related to the underlying neural circuitry which is already different in at-risk individuals as compared to healthy individuals. Furthermore, the absence of behavioural differences between these groups suggests that there was no impairment in the performance of the at-risk adolescents indicating that those adolescents were healthy during the neuroimaging experiment. There was, however, a main effect of face condition, such that all adolescents were less accurate in gender labeling neutral faces. Performance on the out-of-the scanner emotion labeling task also indicated that all adolescents were less accurate in labeling neutral faces relative to fearful and happy faces. These behavioral data are consistent with previous findings that children and adolescents find neutral faces more ambiguous and more difficult to identify than emotional faces [35], [36]. Taken together, these findings suggest that neutral faces may have been more difficult to label in all adolescents in the present study, and that this greater level of difficulty in perceptual discrimination may have warranted greater recruitment of neural regions, including ventromedial prefrontal cortex and superior temporal sulcus, which contributed to the classifier that differentiated the two groups. Moreover, at-risk adolescents may have perceived neutral faces presented in the context of happy faces not only as ambiguous and “non-neutral” but potentially as more threatening than did healthy controls, which would have influenced their pattern of activation in these key neural regions. This interpretation would be consistent with the idea that other emotions can influence the interpretation of neutral faces [37], and may help to explain why groups were classified based on recruitment of key neural regions implicated in face processing and emotion regulation. Nevertheless, including subjective emotional ratings of neutral and other emotional facial expressions in future studies may help elucidate these findings further.

The following limitations to the current study merit some discussion. Although the sample size in the current study was sufficient to provide adequate power to train a GPC to discriminate between adolescents at risk for mood disorders vs. adolescents at low risk, further analyses to distinguish between those who developed vs. those who not develop mood disorder could be conducted only on a subset of the at-risk adolescents who had complete diagnostic interview data. Our findings do, however, provide a rationale for future studies, with larger samples of converters and nonconverters from the at-risk group, to examine the extent to which pattern recognition techniques can identify at the individual level those at-risk adolescents who are most likely to develop in the future different Axis I disorders.

In addition, while the leave-one-out cross-validation is the recommended technique for evaluating classifier performance on small samples due to its almost unbiased estimation of the true error rate it has high variance for small sample sizes. Therefore our results should be validated using independent and bigger samples. Nevertheless, our findings are an important first step toward the ultimate goal of using neuroimaging to help predict future clinical course in at-risk adolescents, and highlight the utility of combining neuroimaging and machine learning techniques to identify neuroimaging measures that may ultimately be able to act as predictors of future onset of psychiatric disorders in at-risk adolescents. We recognize the importance of other risk factors (e.g., psychosocial functioning) in the development of psychiatric disorders such as bipolar disorder. Additionally, it is possible that the environmental effects of being raised by a bipolar parent were a potential confound in the present study. Future studies with larger samples will allow us to integrate these factors and examine these more complex prediction models. In future work we also aim to investigate whether other fMRI tasks could lead to better discrimination between the groups and also strategies to combine different information into the model (e.g. different imaging modalities, clinical and behavioural information).

In summary, our findings indicate that the combination of machine learning and neuroimaging have great potential, especially in situations where there is limited clinical and genetic information, to help to identify which individual at-risk adolescents are at true risk of developing future Axis I disorders. This in turn can help guide early and appropriate interventions for these adolescents and their families, to relieve the significant psychological problems associated with lack of knowledge about the future likelihood of psychiatric disorders in individual at-risk adolescents.

## Supporting Information

Table S1 **Estimated Marginal Means and Standard Errors for Accuracy and Reaction Time Measures for the Happy Face and Fearful Face fMRI Gender-labeling Tasks.** Abbreviations: HBO = healthy offspring having a parent diagnosed with bipolar disorder; HC = healthy control offspring of healthy parents; SE, standard error; RT, reaction times; ms, millisecond; fMRI, functional magnetic imaging. * Significant main effect of face condition for percent accuracy scores (Happy face task: F(2, 29) = 15.8, *p*<.001; Fearful face task: F(2, 29) = 11.23, *p*<.001). Post hoc comparisons indicated Neutral<Happy 100% and Happy 50%, *p*<.05 and Neutral<Fearful 100%, *p*<.05, with a trend for Fearful 50% p = .06.
(DOCX)

Table S2 **Estimated Marginal Means and Standard Errors for Accuracy and Reaction Time Measures for the Post-scanning Emotion Labeling Task.** Abbreviations: HBO = healthy offspring having a parent diagnosed with bipolar disorder; HC = healthy control offspring of healthy parents; SE, standard error; RT, reaction times; ms, millisecond; fMRI, functional magnetic imaging. * There a significant main effect of face condition for accuracy scores, F(2, 22) = 11.1, *p*<.001. Post hoc comparisons indicated Neutral<Fearful and Happy faces, *p*<.05. † There a main effect of face condition for correct-trial reaction times, F(2, 22) = 6.23, *p* = .007. Post hoc comparisons indicated Fearful>Neutral and Happy faces, *p*<.05.
(DOCX)

Table S3 **Most discriminative areas for HBO** ***vs*** **HC.** We listed the regions in the highest weight values, ie, highest contribution for the decision function. The coordinates were obtained using the script 3dclust in AFNI, (http://afni.nimh.nih.gov/pub/dist/doc/manual/3dclust.pdf) and the regions corresponding to the selected coordinates were obtained using the software Tailarach Client.
(DOCX)

Text S1 **Supplemental Information.**
(DOCX)
