# Supplementary material for: Pattern Recognition and Functional Neuroimaging Help to Discriminate Healthy Adolescents at Risk for Mood Disorders from Low Risk Adolescents
Source: PLoS One. 2012 Feb 15;7(2):e29482. doi: 10.1371/journal.pone.0029482 (PMC3280237; doi:10.1371/journal.pone.0029482)
Supplement: Table S1 — Estimated Marginal Means and Standard Errors for Accuracy and Reaction Time Measures for the Happy Face and Fearful Face fMRI Gender-labeling Tasks. Abbreviations: HBO = healthy offspring having a parent diagnosed with bipolar disorder; HC = healthy control offspring of healthy parents; SE, standard error; RT, reaction times; ms, millisecond; fMRI, functional magnetic imaging. * Significant main effect of face condition for percent accuracy scores (Happy face task: F(2, 29) = 15.8, p<.001; Fearful face task: F(2, 29) = 11.23, p<.001). Post hoc comparisons indicated Neutral<Happy 100% and Happy 50%, p<.05 and Neutral<Fearful 100%, p<.05, with a trend for Fearful 50% p = .06. (DOCX) [file pone.0029482.s001.docx]

|  | **Group** | | | | |
| --- | --- | --- | --- | --- | --- |
|  | **HBO**  **(n= 16)** | | | **HC**  **(n = 16)** | |
|  | **Mean** | **SE** | | **Mean** | **SE** |
| **Happy Face Task** |  | |  |  |  |
| *Accuracy,%* |  | |  |  |  |
| Happy 100% | 86.9 | | 3.7 | 83.7 | 2.2 |
| Happy 50% | 87.8 | | 3.7 | 84.7 | 2.1 |
| Neutral^*^ | 82.2 | | 3.5 | 78.0 | 2.5 |
| *Correct-trial RTs, ms* |  | |  |  |  |
| Happy 100% | 923.6 | | 33.5 | 937.7 | 34.8 |
| Happy 50% | 941.7 | | 35.9 | 938.5 | 37.6 |
| Neutral | 925.7 | | 34.0 | 902.8 | 35.5 |
| **Fearful Face Task** |  | |  |  |  |
| *Accuracy,%* |  | |  |  |  |
| Fearful 100% | 87.5 | | 3.8 | 86.0 | 2.3 |
| Fearful 50% | 86.3 | | 4.4 | 84.3 | 3.1 |
| Neutral^*^ | 82.2 | | 3.8 | 82.3 | 2.5 |
| *Correct-trial RTs, ms* |  | |  |  |  |
| Fearful 100% | 965.5 | | 36.4 | 964.4 | 37.6 |
| Fearful 50% | 963.9 | | 39.0 | 954.6 | 40.9 |
| Neutral | 916.9 | | 35.3 | 962.1 | 37.0 |
