# Supplementary material for: Pattern Recognition and Functional Neuroimaging Help to Discriminate Healthy Adolescents at Risk for Mood Disorders from Low Risk Adolescents
Source: PLoS One. 2012 Feb 15;7(2):e29482. doi: 10.1371/journal.pone.0029482 (PMC3280237; doi:10.1371/journal.pone.0029482)
Supplement: Table S2 — Estimated Marginal Means and Standard Errors for Accuracy and Reaction Time Measures for the Post-scanning Emotion Labeling Task. Abbreviations: HBO = healthy offspring having a parent diagnosed with bipolar disorder; HC = healthy control offspring of healthy parents; SE, standard error; RT, reaction times; ms, millisecond; fMRI, functional magnetic imaging. * There a significant main effect of face condition for accuracy scores, F(2, 22) = 11.1, p<.001. Post hoc comparisons indicated Neutral<Fearful and Happy faces, p<.05. † There a main effect of face condition for correct-trial reaction times, F(2, 22) = 6.23, p = .007. Post hoc comparisons indicated Fearful>Neutral and Happy faces, p<.05. (DOCX) [file pone.0029482.s002.docx]

|  | **Group** | | | | |
| --- | --- | --- | --- | --- | --- |
|  | **HBO**  **(n= 16)** | | | **HC**  **(n = 16)** | |
|  | **Mean** | **SE** | | **Mean** | **SE** |
| *Accuracy,%* |  | |  |  |  |
| Overall | 81.1 | | 1.3 | 79.3 | 1.4 |
| Happy | 84.6 | | 4.9 | 93.7 | 5.2 |
| Fearful | 80.8 | | 4.6 | 78.1 | 4.8 |
| Neutral^*^ | 67.3 | | 3.4 | 60.4 | 3.4 |
| *Correct-trial RTs, ms* |  | |  |  |  |
| Overall | 1696.8 | | 224.28 | 1570.0 | 160.0 |
| Happy | 1222.3 | | 117.4 | 1148.9 | 122.2 |
| Fearful^†^ | 1954.7 | | 341.4 | 1815.7 | 355.4 |
| Neutral | 1536.3 | | 160.1 | 1156.7 | 166.6 |
