# Supplementary material for: Pattern Recognition and Functional Neuroimaging Help to Discriminate Healthy Adolescents at Risk for Mood Disorders from Low Risk Adolescents
Source: PLoS One. 2012 Feb 15;7(2):e29482. doi: 10.1371/journal.pone.0029482 (PMC3280237; doi:10.1371/journal.pone.0029482)
Supplement: Table S3 — Most discriminative areas for HBO vs HC. We listed the regions in the highest weight values, ie, highest contribution for the decision function. The coordinates were obtained using the script 3dclust in AFNI, (http://afni.nimh.nih.gov/pub/dist/doc/manual/3dclust.pdf) and the regions corresponding to the selected coordinates were obtained using the software Tailarach Client. (DOCX) [file pone.0029482.s003.docx]

|  | **Region** | **X** | **Y** | **Z** | **Brodmann area** | **Weights** |
| --- | --- | --- | --- | --- | --- | --- |
| **1** | Ventromedial Prefrontal Cortex | 0 | 52.8 | -17.3 | 11 | -33.8 |
| **2** | Superior Temporal Gyrus | -35.3 | 8.5 | -23.2 | 38 | -30.0 |
| **3** | Lingual Gyrus | 5.9 | -88.9 | 0.3 | 17 | -29.6 |
| **4** | Thalamus | 0 | -21 | 20.8 | * | -24.0 |
| **5** | Superior Temporal Sulcus | -50 | 11.5 | -5.6 | 22 | 23.7 |
| **6** | Inferior Occipital Gyrus | -41.2 | -80 | -14.4 | 18 | 21.0 |
| **7** | Superior Parietal Lobule | 38.3 | -71.2 | 44.3 | 7 | -20.9 |
| **8** | Middle Temporal Gyrus | 58.9 | -56.4 | 6.2 | 21 | 19.9 |
| **9** | Inferior Temporal Gyrus | -61.8 | -6.2 | -14.4 | 21 | -19.8 |
| **10** | Middle Frontal Gyrus | 44.2 | 20.3 | 20.8 | 46 | -18.4 |
| **11** | Ventromedial Prefrontal Cortex | 0 | 29.2 | -14.4 | 11 | 17.7 |
| **12** | Superior Temporal Gyrus | -47.1 | 14.4 | -14.4 | 38 | -17.3 |
| **13** | Culmen | -17.7 | -35.8 | -23.2 | * | -17.1 |
| **14** | Precuneus | 2.9 | -50.5 | 38.4 | 7 | 17.0 |
| **15** | Postcentral Gyrus | 61.8 | -24 | 20.8 | 40 | 16.9 |
