# Supplementary material for: Pattern Recognition and Functional Neuroimaging Help to Discriminate Healthy Adolescents at Risk for Mood Disorders from Low Risk Adolescents
Source: PLoS One. 2012 Feb 15;7(2):e29482. doi: 10.1371/journal.pone.0029482 (PMC3280237; doi:10.1371/journal.pone.0029482)
Supplement: Text S1 — Supplemental Information. (DOCX) [file pone.0029482.s004.docx]

SUPPLEMENTAL INFORMATION

Image acquisition

Neuroimaging data were collected using a 3T Siemens Allegra MRI scanner at the University of Pittsburgh/Carnegie Mellon University Brain Imaging Research Center. Structural 3D Sagittal MPRAGE images were acquired with the following parameters: 6 min.7 sec., TE: 2.48 ms, TR: 1630 ms, IT: 800ms, flip angle: 8°, field of view: 200 mm, slice thickness: 0.8 mm, image matrix: 256 x 256, 208 slices. Mean blood-oxygenation-level-dependent (BOLD) images were acquired with a reverse gradient-echo EPI sequence: 34 axial slices (3mm thick, 0mm gap; TR/TE=2000/25msec, FOV=205 mm, matrix=64x64), parallel to the AC-PC line and encompassing the entire cerebrum and the majority of the cerebellum. Participants were placed in the simulator prior to scanning to help them habituate to the scanning environment and minimize head movement.

Data preprocessing and analysis

Data preprocessing was performed using SPM5 (http://www.fil.ion.ucl.ac.uk/spm). Functional data for each participant were first corrected for differences in acquisition time between slices, realigned using the first slice as a reference, and unwarped to correct for static inhomogeneity of the magnetic field and movement by inhomogeneity interactions. Each volume was co-registered by aligning the first scan from each volume to the first scan of the first volume with regard to the subject's MPRAGE image and segmented. Data were normalized to Montreal Neurological Institute standardized template and spatially smoothed with a Gaussian kernel of 6-mm full-width at half-maximum.

fMRI Paradigm

An emotional face gender labeling event-related fMRI paradigm was used. It comprised two, well-validated 6-minute fast event-related neuroimaging tasks examining neural activity to happy versus neutral (happy face task) and fearful versus neutral (fearful face task) emotional facial expressions. All stimuli were gray-scale digitized photographs from that were of fixed size (15×10.5 cm), cropped, and morphed using software to depict emotional expressions ranging from neutral (0%) to mild (50%) to prototypical (100%) intensity of each emotion. Each stimulus was presented for 2 sec. with a mean inter-stimulus interval of 4.9 sec. during which a fixation cross was displayed. In each experiment, participants viewed 20 neutral, 20 mild, and 20 prototypical faces. The order of the two tasks was counterbalanced. Subjects were asked to respond with their index finger or the middle finger to indicate whether the actor in the picture was a woman or a man. They were also asked to try to respond as quickly but also as accurately as possible.

Clinical Self-report and IQ Measures

Parents completed the following questionnaires about their children: the StonyBrook Child or Adolescent Symptom Inventory-4 [1[, to assess for DSM-IV Axis I diagnoses; the Mood and Feelings Questionnaire (MFQ) [2], to assess for symptoms of depression; the Child Affect Lability Scale (CALS) [3], to assess for mood lability, and the Screen for Childhood Anxiety and Related Disorders (SCARED) [4] to assess for symptoms of anxiety. Offspring completed the child self-report version of the MFQ and SCARED. Socio-economic status (SES) was measured with the Hollingshead Four-Factor Index [5]. IQ was determined using the Wechsler Abbreviated Scale of Intelligence (WASI)[6].

Supplemental references:

1. Gadow KD, Sprafkin J (1987) Discriminant validity of a DSM-III-based checklist. Reported in Gadow and Sprafkin (1994), Child Symptom Inventories manual. Stony Brook, NY: Checkmate Plus.
2. Angold A, Costello EJ, Pickles A, Winder F, Silver D (1987) The development of a questionnaire for use in epidemiological studies of depression in children and adolescents. London: Medical Research Council Child Psychiatry Unit.
3. Gerson AC, Gerring JP, Freund L, Joshi PT, Capozzoli J, Brady K, Denckla M B (1996) The Children's Affective Lability Scale: a psychometric evaluation of reliability. Psychiatry Research 65: 189-198.
4. Birmaher B, Ketharpal S, Brent D, Cully M, Balach M, Kaufman J, et al. (1997) The screen for child anxiety related emotional disorders (SCARED): Scale construction and psychometric characteristics. Journal of the American Academy of Child and Adolescent Psychiatry 36: 545-553.
5. Hollingshead AB (1975) Four Factor Index of Social Status. (New Haven, CT: Yale University Department of Sociology.

6. *Wechler Abbreviated Scale of Intelligence (WASI)*: Psychological Corporation; 1999.
